# Pathological Spectrum of Ovine Pulmonary Adenocarcinoma in Small Ruminants: A Focus on the Mixed Form

**DOI:** 10.3390/ani13182828

**Published:** 2023-09-06

**Authors:** Joaquín Ortega, Juan M. Corpa, Diego Castillo, Brian G. Murphy

**Affiliations:** 1 Pathology Group, PASAPTA, Facultad de Veterinaria, Universidad CEU Cardenal Herrera, CEU Universities, Av. Seminario s/n, Moncada, 46113 Valencia, Spain; jmcorpa@uchceu.es; 2 Department of Pathology, Microbiology and Immunology, School of Veterinary Medicine, University of California, Davis, CA 95616-5270, USA; ldcastillo@ucdavis.edu (D.C.); bmurphy@ucdavis.edu (B.G.M.)

**Keywords:** sheep, JSRV, jaagsiekte, ovine pulmonary adenocarcinoma

## Abstract

**Simple Summary:**

This paper describes pathological findings in small ruminants with ovine pulmonary adenocarcinoma, a contagious respiratory tumor caused by the jaagsiekte sheep retrovirus. Infection with JSRV leads to the formation of lung tumors, progressive respiratory failure and, ultimately, the death or culling of the affected animals. OPA has traditionally been divided into two forms: classical and atypical. However, in this study, we examined 27 animals with OPA, and found that most of the observed tumors displayed a mix of characteristics from both the classical and the atypical form, and were classified as mixed. Grossly, the lesions were found mostly in the cranial lobes of the lungs, appearing as firm, flat-to-slightly-raised masses that varied in size and color. Histologically, the cases were categorized based on the predominant architectural patterns, with the mixed pattern being the most common. The objective of this study was to provide a detailed description of the gross and microscopic spectrum of ovine pulmonary adenocarcinoma in small ruminants from Spain, focusing on the mixed form. It is important to note that the mixed form of OPA is less frequently reported, and can be mistaken for other concurrent lung diseases.

**Abstract:**

Ovine pulmonary adenocarcinoma (OPA) is a contagious respiratory tumor of small ruminants, manifesting in chronic weight loss and respiratory failure. Infection with the betaretrovirus jaagsiekte sheep retrovirus (JSRV) is the cause of OPA. Here, we describe the gross and microscopic features of twenty-six sheep and one goat with naturally occurring JSRV-associated OPA. All the animals included in this study had pulmonary lesions morphologically consistent with OPA, but the majority of the observed lesions demonstrated features of both the classical and the atypical form of OPA, and were, therefore, classified grossly as mixed. The gross lesions were located mainly in the cranial pulmonary lobes, were multifocal to coalescing, variable in number and size, flat to slightly raised, firm, and white to grey. Histologically, the cases were classified according to the predominant architectural patterns as lepidic, papillary, acinar, or mixed; the mixed histological pattern was the most prevalent. The aim of this study was to describe the gross and microscopic spectrum of OPA in naturally infected small ruminants from Spain. The mixed form of OPA is less commonly reported, and can be confused with other concurrent pulmonary pathologies (such as BALT hyperplasia in SRLV-associated pneumonia or lungworm granulomas).

## 1. Introduction

Ovine pulmonary adenocarcinoma (OPA), also referred to as ovine/sheep pulmonary adenomatosis, is a contagious viral disease in sheep and, less often, goats that results in the formation of tumors in the lower respiratory tract [1,2,3]. OPA occurs as a result of infection with the betaretrovirus jaagsiekte sheep retrovirus, or JSRV [1,2,3]. *Jaagsiekte* is the Afrikaans name for OPA, and was first recognized as a disease in South African sheep during the 19th century [1,2,3]. OPA is commonly diagnosed in the United Kingdom, South America, and South Africa, occurs regularly in multiple European countries, and is endemic in Scotland and Spain [1,2,3]. A study in a Spanish abattoir recorded visible OPA lesions in up to 1.4% of sheep slaughtered over a year [3]. OPA has not been identified in Australia or New Zealand [3], and is rarely diagnosed in North American sheep, where it is considered to be a sporadic disease of low incidence [1,4]. The marked geographic variation in the occurrence of OPA may be the result of multiple factors, including differences between rates of infection in different countries, sequence variations in the circulating viral strains, variations in the host ruminant susceptibility, or may simply reflect a lack of exposure [5]. OPA was eradicated from Iceland in 1952 using extreme depopulation measures requiring the slaughter of 600,000–700,000 sheep [4].

Clinically, OPA is most often diagnosed in sheep (and rarely in goats) of 1–4 years of age, and typically features one or more variably sized, generally well-circumscribed pulmonary adenocarcinomas, associated with progressive dyspnea, anorexia, weight loss, and emaciation [1,3]. Due to their clinical and pathological overlap, OPA can be challenging to distinguish from pneumonia resulting from an infection with small ruminant lentivirus (SRLV) [4]. In rare circumstances, OPA tumor metastases have been documented in the thoracic lymph nodes, liver, kidney, heart, skeletal muscle, digestive tract, spleen, skin, or adrenal glands [5,6,7]. The spontaneous regression of pulmonary tumors has also been documented in experimentally infected lambs, and is suspected to be the result of a T-cell-mediated immune response [8].

As the pulmonary tumor burden increases, the production of surfactant-containing fluid by the transformed epithelial cells increases, resulting in copious pulmonary effusion, nasal discharge, and progressive dyspnea, which can be accentuated by exercise [4,9]. During the final stage of the disease, up to 400 mL of respiratory tract fluid may be discharged from the affected animal’s nostrils when the hindquarters are elevated (the so-called “wheel barrow test”) [3]. OPA eventually results in the death or culling of the affected sheep and, as a result, can have a significant economic impact on the sheep industry [3].

Grossly, two different forms of OPA are known to occur, the classical and the atypical, although mixed or intermediate forms of OPA have also been cited [6,10]. In the classical form of OPA, the lungs fail to collapse when the thorax is initially opened, and the cranioventral lung is typically the most severely affected [3]. Affected lungs have variably sized, coalescing neoplastic nodules, associated with regions of pulmonary atelectasis [4], and the tumors fail to protrude from the cut surface of the lung [3]. Affected lungs are heavy, and may exude clear fluid on sectioning; additional frothy fluid may be present in the bronchi and trachea, and concurrent pneumonia is often present [4]. The atypical form of OPA features well-demarcated, solitary, or multifocal nodules, distributed primarily in the caudal pulmonary lobes [3,10]. The neoplastic foci in the atypical form of OPA are more solid than in the classical form, and tend to exude less fluid [4,10]. Histologically, OPA lesions are consistent with a well-differentiated, multicentric, bronchioloalveolar carcinoma (adenocarcinoma) [4]. The pulmonary tumors are characterized histologically as a proliferation of well-differentiated cuboidal-to-columnar epithelial cells. In some lesions, the associated pulmonary interstitium may have significant collagen deposition and overt fibrosis [4], with occasional myxoma-like nodules [11].

The aim of this study was to describe the pathological spectrum of OPA in naturally infected small ruminants, focusing on the morphological features of the less-commonly reported mixed lesions. It has been described that the classical and the atypical form of OPA likely represent two extremes of a disease spectrum, rather than separate disease entities [11]. Here, we describe the gross and microscopic features of naturally occurring OPA lesions in small ruminants from Spain. Most of these lesions did not fit into either the classical or atypical gross classification of OPA, and were determined to be mixed.

## 2. Materials and Methods

### 2.1. Animals

A search was conducted in the Veterinary Pathology Diagnostic Service of the Universidad CEU Cardenal Herrera (Valencia, Spain) from 2015 to 2022 for small ruminant submissions diagnosed with ovine pulmonary adenocarcinoma. A complete necropsy was performed for each animal, and the gross lesions were described and photographed. Selected tissues were fixed in 10% buffered formalin for a maximum of 48 h, trimmed, embedded in paraffin, routinely processed for histological examination, and stained with hematoxylin and eosin. All of the tissues were examined by board certified veterinary pathologists. Formalin fixed paraffin embedded (FFPE) tissue blocks from Spain were mailed to UC Davis, for further molecular analyses.

### 2.2. Molecular Testing for Lesion-Associated Exogenous JSRV

Genomic DNA was isolated from formalin-fixed, paraffin-embedded blocks of pulmonary tissues, and hemi-nested PCR was performed for the determination of exogenous JSRV, essentially as described previously [12]. DNA was isolated from 5 × 10 μm thick paraffin curls, using the QIAamp DNA FFPE Tissue Kit (Qiagen, Germantown, MD, USA), according to the manufacturer’s instructions. An initial round of amplification was performed using the P5 and P2 primers previously described by Palmarini et al. [12] Primers P5 (5′-TGG GAG CTC TTT GGC AAA AGC C) and P2 (5′-ATA CTG CAG CYC GAT GGC CAG) target a ~2064 base pair region of the proviral LTR and *gag* gene. Pfu Turbo DNA Polymerase (Agilent Technologies, Santa Clara, CA, USA) was used under the following cycling conditions; 95 °C for 2 min, followed by 35 cycles of 95 °C for 30 s, 55 °C for 30 s, 72 °C for 2.5 min, and a final elongation step of 72 °C for 10 min.

Five microliters from the initial reaction were carried over into a hemi-nested PCR reaction, using the primers P1 (5′-GCT GCT TTR AGA CCT TAT CGA AA) and P2 (above), in order to amplify a 229 bp region within *gag* using Q5 High-Fidelity DNA Polymerase (New England Bio Labs, Ipswich, MA, USA), under the following cycling conditions: 98 °C for 30 s, followed by 35 cycles of 98 °C for 10 s, 63 °C for 30 s, 72 °C for 30 s, and a final elongation step of 72 °C for 2 min. Plasmid DNA encoding the JSRV LTR and gag gene (positive control) and water template (negative control) PCR reactions were run in parallel in both the first and second amplifications.

The resulting amplicons were visually assessed for ~230 bp amplicon, using ethidium bromide agarose gel electrophoresis.

### 2.3. Small Ruminant Lentivirus (SRLV) Testing

All of the cases were tested for concurrent maedi-visna virus (MVV) infection using a combination of standard PCR-based techniques and serology (ELISA). Note: MVV is also referred to as small ruminant lentivirus, or SRLV. Standard PCR was performed using published primers in all cases, in an attempt to amplify an approximately 300 bp region of the MVV LTR, using the following primer set: MVV LTR for 2 (5′-ACT GTC AGG RCA GAG AAC ARA TGC C), and MVV LTRrev2 (5′-CCA CGT TGG GCG CCA GCT GCG AGA), under the following cycling conditions: 95 °C for 3 min, followed by 40 cycles of 95 °C for 30 s, 64 °C for 30 s, 72 °C for 1 min, and a final elongation of 72 °C for 5 min [13]. The resulting PCR amplicons were assessed for appropriate size (~300 bp), using agarose gel electrophoresis.

In addition, assessment of the SRLV serologic status in antemortem serum was performed using the IDEXX MVV/CAEV p28 Ab Screening Test (IDEXX Laboratories, Westbrook, ME, USA), according to the manufacturer’s instructions.

## 3. Results

### 3.1. Pathology

From 2015 to 2022, 1276 small ruminants from various flocks in the Valencian Community (eastern Spain) were submitted to the Veterinary Pathology Diagnostic Service for necropsy. OPA was diagnosed in 27 animals (2.1%) based on gross, histologic, and molecular findings. All were adults and female. The signalment, pathological, and molecular findings are summarized in Table 1.

**Figure 1:**
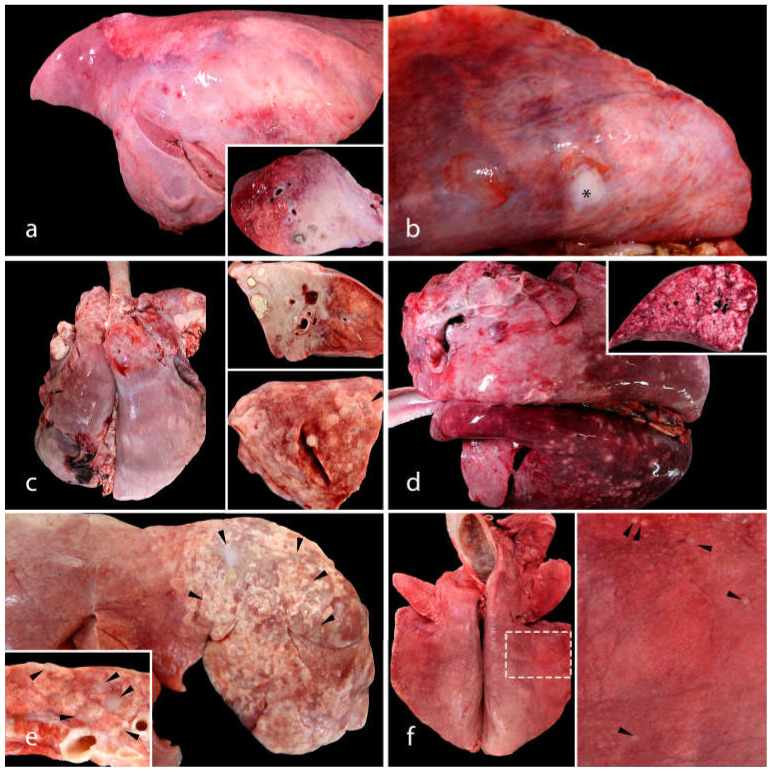
The gross pulmonary lesions of OPA. (**a**) The classical form of OPA in a sheep (case 12). The cranioventral areas of the lung (up to 70–80% of the pulmonary parenchyma) are replaced by a firm, white-to-light-purple mass. On the cut surface, there is a diffuse firm area, with several spaces filled with necrotic material. (**b**) The atypical form of OPA in a goat (case 3). A solitary, white, firm, raised nodule is observed in the caudal right lobe (asterisk). (**c**–**f**) Mixed forms of OPA in sheep. (**c**) Different OPA lesions in the cranial lobes of the same lung (case 15): diffuse white and firm areas with necrotic material (top right), and multiple white-to-grey well-demarcated nodules (bottom right). (**d**) The cranial portion of this lung presents lesions consistent with the classical form of OPA (diffuse, firm, pink areas) and the caudal portion with the atypical presentation (multifocal, white, firm, raised nodules) (case 21). (**e**) Numerous grey-to-white flat OPA lesions (case 4) in the right cranial lobe (arrowheads). (**f**) Multifocal pinpoint (1–2 mm in diameter) white lesions (case 5) of OPA (arrowheads).

The location, severity, and morphology of the lesions were highly variable. Only three cases (case 9, 12 and 19) had lesions grossly consistent with the classical form, and two cases (case 1 and 3 (the goat)) demonstrated lesions characteristic of the atypical form. In the classical OPA lesions, the neoplastic lesions were in the cranioventral portions of the lung, were white or light purple, and did not protrude from the cut surface of the lung (Figure 1a). On the cut section, the pulmonary lesions were diffusely white and firm, and cavities containing necrotic-to-purulent material were frequently observed. The two cases of atypical OPA each presented with a solitary, white, firm, raised nodule in the caudal right lobe (Figure 1b). The gross lesions in the 22 remaining sheep shared features of both the classical and the atypical forms of OPA and were, therefore, classified as mixed (Figure 1c,d). These mixed lesions were located mainly in the cranial pulmonary lobes, were multifocal to coalescing, flat to slightly raised, firm, white to grey, and variable in size (up to 3 cm in diameter), and had a dry cut surface. Some of the gross lesions were very small (1–2 mm diameter), subtle, and poorly delineated (Figure 1e,f).

**Figure 2:**
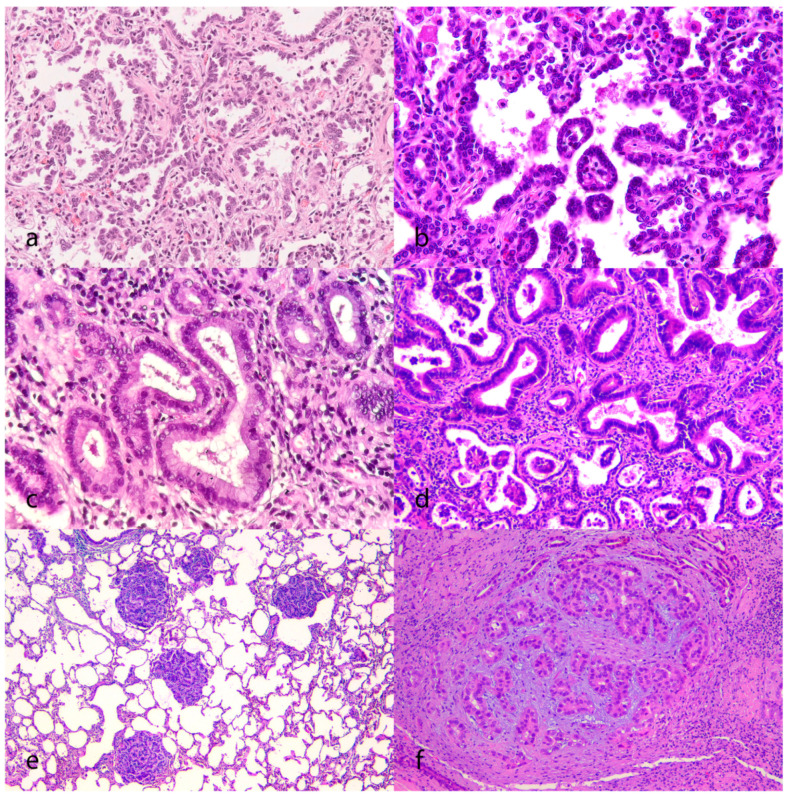
The histological patterns of OPA. (**a**) The lepidic pattern is characterized by palisading cuboidal epithelial monolayers lining alveolus-like structures (case 12). 200×, HE. (**b**) The papillary pattern features exophytic-to-polypoid growths of cuboidal-to-columnar cells, arranged on the surfaces of the fibrovascular core (case 11). 200×, HE. (**c**) The acinar pattern features palisades of taller columnar epithelial cells that form tubular structures (case 14). 200×, HE. (**d**) The mixed pattern, demonstrating tall columnar cells (top of image) and low cuboidal (bottom) cells arranged in different patterns (case 14). 200×, HE. (**e**) Multiple small nodules composed of tightly packed neoplastic cuboidal cells (histology from the lung in Figure 1f) (case 5). 40×, HE. (**f**) A nodule composed of neoplastic cells associated with a moderate amount of basophilic extracellular matrix, with myxoid differentiation (case 1). 100×, HE.

Microscopically, the OPA lesions were classified according to their architectural patterns as lepidic, papillary, acinar, or mixed (Table 1 and Figure 2). The lepidic subtype (3 cases) was characterized by a layer of cuboidal epithelium following an alveolar pattern (Figure 2a). The papillary subtype (six cases) presented exophytic growths of cuboidal-to-columnar cells arranged around a fibrovascular core (Figure 2b). The acinar subtype (5 cases) was characterized by palisades of taller columnar cells that formed tubular structures often surrounded by fibrovascular stroma (Figure 2c). Some of the acinar structures showed mucous differentiation. Mixed patterns were the most frequently identified (13 cases), and they were composed of a combination of the previously described subtypes or the intermediate forms between them (Figure 2d). In sheep with subtle 1–2 mm diameter pulmonary lesions (the mixed form of OPA), the diagnosis of OPA was only confirmed after histological examination (Figure 2e). In several cases, proliferative pulmonary nodules were composed of neoplastic cells associated with a moderate amount of extracellular matrix, with myxoid differentiation (fibromyxoid nodules, Figure 2f).

In addition to the OPA lesions, all the animals had some form of concurrent pneumonia of varying severity: either broncho-, interstitial, bronchointerstitial, or granulomatous pneumonia. The additional pulmonary lesions that were identified included broncho-associated lymphoid tissue (BALT) hyperplasia, tumor-associated fibromyxoid nodules, pulmonary abscesses, and pulmonary nematode infestations (lungworms, Table 1). Several of these lesions have been previously identified and described in sheep with JSRV-associated OPA [10]. Nodules associated with lungworms were present in several cases (*n* = 10), and were grossly characterized by numerous, grey-to-green, firm parenchymal granulomas, located mostly in the caudodorsal aspect of the lung. Histologically, sections of nematode eggs and larvae and, occasionally, adults were associated with the granulomatous inflammation. The identification of the nematode species present in each lung was not possible to perform on the histological sections. However, parasitological studies conducted on sheep from the same flocks demonstrated that the most common species of lungworms in our area were *Muellerius capillaris*, *Neostrongylus linearis*, and *Cystocaulus ocreatus.*

### 3.2. Molecular Testing for Exogenous JSRV

DNA isolated from the pulmonary lesions of all cases yielded appropriately sized PCR amplicons (~230 bp) consistent with the presence of exogenous JSRV [12]. Positive (plasmid DNA) and negative (water template) controls, run in parallel, were appropriate.

### 3.3. MVV / SRLV Results

Two of the cases were determined to be positive for concurrent SRLV using MVV PCR (cases 14 and 15), and two were positive using serology (cases 25 and 27). The control samples run in parallel were appropriate. Cases 25 and 27 were serologically positive for SRLV, and PCR-negative for intralesional MVV. The sensitivity of the p28 antibody screening test (serology) is likely superior to the PCR test, as PCR sensitivity is highly sequence-dependent, and the formalin fixation of the tissue may have reduced the sensitivity of the PCR assay.

## 4. Discussion

It has been stated that the classical and the atypical form of OPA likely represent two extremes of a disease spectrum, rather than separate disease entities [10]. Although mixed or intermediate forms of OPA have been cited in several reports, there is limited descriptive information about the gross and histological features of these presentations [3,6,10].

All the animals in this study had pulmonary lesions that were grossly and histologically consistent with OPA, and were confirmed to have lesion-associated exogenous JSRV via hemi-nested PCR testing. Animals with the classical form (i.e., tumors affecting large areas of the cranioventral portions of the lung lobes) and atypical form (solitary, pearly white, raised, firm nodules located mostly in the caudal lobes) of OPA demonstrated gross lesions consistent with those previously reported [6,10]. However, most of the animals (22/27, 81%) demonstrated gross lesions consistent with the mixed form of OPA. The overall abundance of mixed-form lesions in this cohort of animals differs from previous reports, where up to 88% of the cases presented with the classical form [11]. In several cases, the gross OPA lesions were very small (1–2 mm). This may be because the OPA lesions were identified as incidental findings during the early stage of the disease (they were culled animals, with the presence of other concurrent, and more severe, diseases).

Previous reports stated that the histological appearance of atypical OPA is essentially the same as that of classical OPA, but the pattern of epithelial neoplasia is more often acinar than papillary, and the stroma is more heavily infiltrated by inflammatory cells and connective fibers (fibrosis) [10]. In this study, the tumors were histologically classified as lepidic, papillary, acinar, or mixed, according to a recent classification of lung tumors in domestic animals [4]. Although the case numbers were not sufficient to draw meaningful statistical conclusions, no association between the histological pattern and gross presentation of OPA was identified.

It is possible that the JSRV genotype could influence the severity of clinical disease, lesion morphology, and lesion classification. This hypothesis could be tested through obtaining OPA lesion samples at the time of necropsy in an RNA-protected state (lesions preserved in RNAlater), and then performing RT PCR amplification and sequencing. An association between the different lesions and the viral genotype could then be assessed.

OPA-affected lungs in some of the animals also had one or more fibro-myxoid nodules identified histologically. These OPA-associated lesions have been previously described, and the pathogenesis is currently unknown [11].

All the OPA lesions were associated with variable degrees of pulmonary inflammation, including bronchopneumonia, interstitial pneumonia, broncho-interstitial pneumonia, granulomatous pneumonia, and/or pulmonary abscesses. OPA-associated inflammation may be the result of a disruption in the pulmonary defense mechanisms (e.g., the mucociliary escalator, which is responsible for moving mucus up and out of the lower respiratory tract), and the resulting secondary microbial colonization. However, it is also true that co-infections with other pathogens, such as lungworms, small ruminant lentivirus (MVV/CAEV), or multisystemic inflammatory diseases, were identified in multiple animals. In some cases, these lentivirus-associated hyperplastic lesions can be a challenge to microscopically distinguish from the lepidic form of OPA [4]. Hyperplasia of the bronchiole-associated lymphoid tissue (BALT) was identified in multiple animals. This immunologic response is also common in animals infected with SRLV, and was identified in all four of the animals with this coinfection (cases 14, 15, 25, and 27). Co-infections of small ruminants with both JSRV and SRLV have been described previously, possibly because SRLV seems to replicate more readily in sheep with OPA than in animals free of OPA [14]. This has been hypothesized to be the case because of the increased numbers of alveolar macrophages (SRLV target cells) associated with OPA lesions [14].

## 5. Conclusions

The classical and atypical OPA gross lesions are well described in the peer-reviewed literature. They are easier to recognize than OPA mixed lesions, which often feature very small lesions, posing a challenge for the veterinary diagnostician, as they may remain undetected or misdiagnosed with other pathologies (e.g., BALT hyperplasia in SRLV-associated pneumonia, lungworm granulomas, etc.). In such cases, molecular or immunological testing for the intralesional presence of exogenous JSRV provirus may be warranted.

## Figures and Tables

**Table 1:** The signalment, pathological, and molecular findings for a small ruminant with OPA.

Case #	Signalment	Gross	Histology	Other Pulmonary Findings	JSRV Hemi-Nested PCR	SRLV PCR	SRLV Serology	Additional Findings
1	Sheep	A	P ACA (M)	GP, LW, BALT, FMN	+	−	ND	Suppurative arthritis
2	Sheep	M	P ACA (Ac)	IP, BALT	+	−	ND	*D. dendriticum*
3	Goat	A	P ACA (Ac)	BP	+	−	ND	*D. dendriticum*
4	Sheep	M	P ACA (M)	GP, LW, BALT	+	−	ND	
5	Sheep	M	P ACA (L)	BIP	+	−	ND	*D. dendriticum*, *Sarcocystis* spp.,
6	Sheep	M	P ACA (M)	BIP, A	+	−	ND	
7	Sheep	M	P ACA (M)	GP, BALT, LW	+	−	ND	*C. tenuicollis*
8	Sheep	M	P ACA (M)	GP, LW, BALT	+	−	ND	Suppurative arthritis
9	Sheep	C	P ACA (M)	BP, BALT	+	−	ND	
10	Sheep	M	P ACA (L)	BIP	+	−	ND	Hepatic abscesses, *D. dendriticum*, caseous lymphadenitis
11	Sheep	M	P ACA (P)	IP, MN, BALT, FMN, LW	+	−	ND	Hydatid cysts, *D. dendriticum*, *Sarcocystis* spp., uterine torsion with rupture and peritonitis
12	Sheep	C	P ACA (L)	BIP, A	+	−	ND	Caseous lymphadenitis
13	Sheep	M	P ACA (Ac)	GP, LW, A	+	−	ND	*D. dendriticum*
14	Sheep	M	P ACA (Ac)	GP, BALT, LW, A	+	+	ND	*D. dendriticum*
15	Sheep	M	P ACA (M)	BIP, BALT, FMN	+	+	ND	*C. tenuicollis*, *Sarcocystis* spp.,
16	Sheep	M	P ACA (M)	BP, GP, BALT, LW	+	−	ND	*O. ovis*, *Teladorsagia* sp. *D. dendriticum*
17	Sheep	M	P ACA (M)	BIP/GP, LW	+	−	ND	*D. dendriticum*
18	Sheep	M	P ACA (P)	IP, BALT, IF	+	−	ND	Necrotizing pneumonia, renal amyloidosis, *D. dendriticum*
19	Sheep	C	P ACA (P)	IP, BALT	+	−	ND	*D. dendriticum*
20	Sheep	M	P ACA (Ac)	BIP/GP, LW, BALT, IF	+	−	ND	Necrotizing mastitis
21	Sheep	M	P ACA (M)	BP	+	−	ND	*D. dendriticum*
22	Sheep	M	P ACA (M)	BP, IF, A, BALT	+	−	−	Portal hepatitis *D. dendriticum*
23	Sheep	M	P ACA (P)	BIP, BALT, FMN	+	−	−	*D. dendriticum*
24	Sheep	M	P ACA (M)	BIP, BALT, IF	+	−	−	Portal hepatitis, /cholangitis, *D. dendriticum*, renal amyloidosis,
25	Sheep	M	P ACA (P)	IP, IF	+	−	+	*D. dendriticum*
26	Sheep	M	P ACA (P)	IP, A	+	−	−	Intravascular pulm metastases
27	Sheep	M	P ACA (M)	BIP, BALT, IF	+	−	+	*C. tenuicollis*, *D. dendriticum*

Abbreviations: C, classical; A, atypical; M, mixed; P ACA, pulmonary adenocarcinoma; (L), lepidic; (P), papillary; (Ac), acinar; (M), mixed; A, pulmonary abscesses; BALT, broncho-alveolar lymphoid tissue hyperplasia; BIP, bronchointerstitial pneumonia; BP, bronchopneumonia; GP, granulomatous pneumonia; IP, interstitial pneumonia; FMN, fibromyxoid nodules; LW, lungworms; ND, not determined; *C. tenuicollis*, *Cysticercus tenuicollis*; *D. dendriticum*, *Dicrocoelium dendriticum*; *O. ovis*, *Oestrus ovis.*

## Data Availability

The data reported in this paper were generated specifically for the study, and they are shown in Table 1.
